# Primary angiosarcoma of the small intestine with metastasis to the liver: a case report and review of the literature

**DOI:** 10.1186/1477-7819-11-242

**Published:** 2013-09-25

**Authors:** Qingqiang Ni, Dong Shang, Honghao Peng, Manish Roy, Guogang Liang, Wei Bi, Xue Gao

**Affiliations:** 1 Department of General Surgery, First Affiliated Hospital, Dalian Medical University, Dalian, Liaoning 116011, P.R. China; 2 Department of General Surgery, Linyi Economic And Technological Development Zone People's Hospital, Linyi, Shandong 276000, P.R. China; 3 Department of Surgery, Kathmandu Medical College, 184 Baburam Acharya Sadak, Sinamangal, P.O.Box: 21266, Kathmandu, Nepal; 4 Department of Pathology, First Affiliated Hospital, Dalian Medical University, Dalian, Liaoning 116011, P.R. China

**Keywords:** Small intestine, Primary angiosarcoma, Hepatic metastasis

## Abstract

Angiosarcoma is a rare disease with a poor prognosis; significantly, patients with intestinal angiosarcomas who survive over 1 year after diagnosis are extraordinarily rare. This article describes the case of a 33-year-old gentleman who presented with abdominal pain of 4 months duration, which had increased in severity 2 weeks prior to presentation. After a complicated diagnostic and therapeutic process, the diagnosis of primary angiosarcoma of the small intestine with metastasis to the liver was made by pathological and immunohistochemical examinations. We reviewed previous cases of angiosarcoma described in the English literature to determine their risk factors, diagnosis and treatment, and we found that angiosarcoma is extremely rare, especially in the small intestine. To the best of our knowledge, this may be the youngest case of primary angiosarcoma of the small intestine with metastasis to the liver reported in the English literature.

## Background

Angiosarcomas, which account for only 1 to 2% of all soft tissue sarcomas, are rare malignant tumors of endothelial origin [1,2]. These tumors are usually found in the scalp and facial skin of elderly individuals, with a male preponderance [3]. Primary angiosarcoma of the small intestine is extremely rare; moreover, due to the difficulty of prompt and accurate diagnosis, its prognosis is very poor [1,4]. To the best of our knowledge, few cases of primary angiosarcoma of the small intestine have been reported in the English literature [5], especially primary angiosarcoma of the small intestine with metastasis to the liver in a young patient.

Here, we report a 33-year-old gentleman who presented with abdominal pain of 4 months duration, which worsened in the last 2 weeks prior to presentation.

## Case presentation

A 33-year-old gentleman admitted to our hospital on 5 August 2011 presented with a 4-month history of pain in the left lower quadrant of the abdomen, which had worsened in the last 2 weeks before presentation.

Four months ago, the patient began experiencing abdominal pain in the lower left quadrant without any apparent cause. The symptoms became increasingly aggravated during the last 2 weeks before presentation. The presenting symptoms included paroxysmal colic in the left lower quadrant of the abdomen, fever, nausea and vomiting. The patient also experienced fatigue and an 8 kg weight loss over the last 4 months. In a nearby local hospital, an abdominal plain film was obtained, which showed intestinal tympanites.

**Figure 1:**
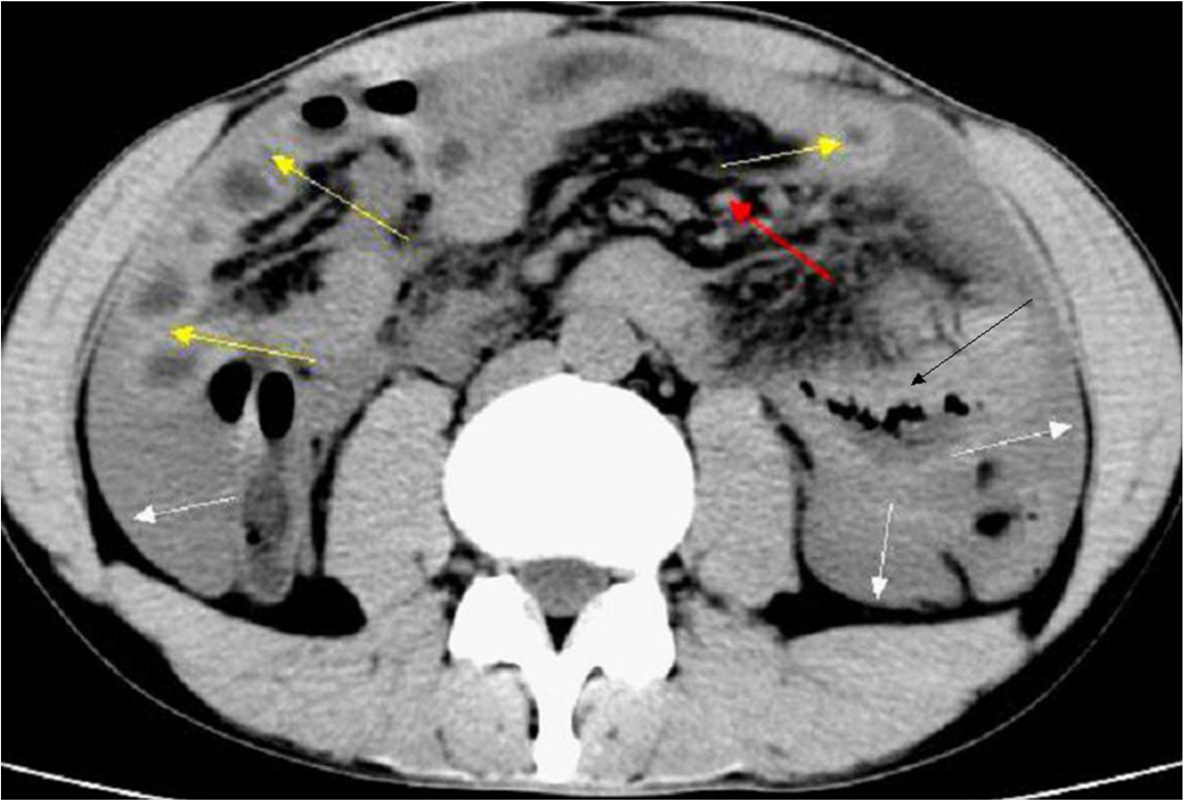
Computed tomography scans of the whole abdomen showed a significantly thickened intestinal wall (yellow arrows) located at the end of the jejunum and the proximal ileum, excessive ascites (white arrows), a few enlarged lymph nodes in the abdomen (red arrow) and the location of the primary angiosarcoma of the small intestine (black arrow).

**Figure 2:**
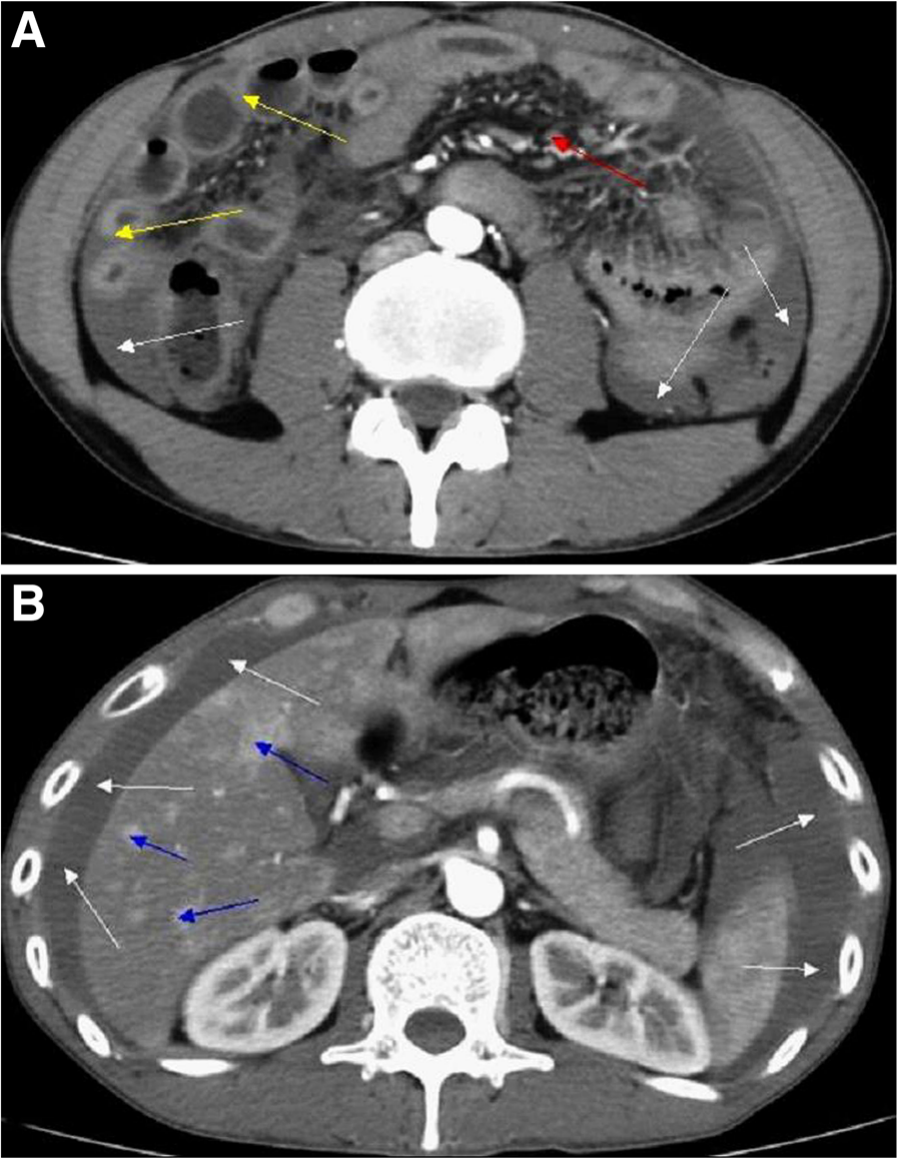
**Enhanced computed tomography scan. (A)** A significantly thickened intestinal wall (yellow arrows) located in the end of the jejunum and the proximal ileum, excessive ascites (white arrows) and a few enlarged lymph nodes in the abdomen (red arrow). **(B)** Different degrees of enhancement were noted in the hepatic arterial phase (blue arrows).

For further treatment, the patient came to our outpatient department and was admitted to the Department of Gastroenterology. A physical examination revealed a male patient with tenderness in the left lower abdominal region. There was no hepatomegaly or splenomegaly. Laboratory tests were performed, and the results were normal, including the levels of carcinoembryonic antigen and carbohydrate antigen 19–9. Computed tomography (CT) scans of the whole abdomen showed a significantly thickened intestinal wall located at the end of the jejunum and the proximal ileum, excessive ascites and a few enlarged lymph nodes in the abdomen (Figure 1). Contrast-enhanced CT scans showed different degrees of enhancement in the hepatic arterial phase (Figure 2).

**Figure 3:**
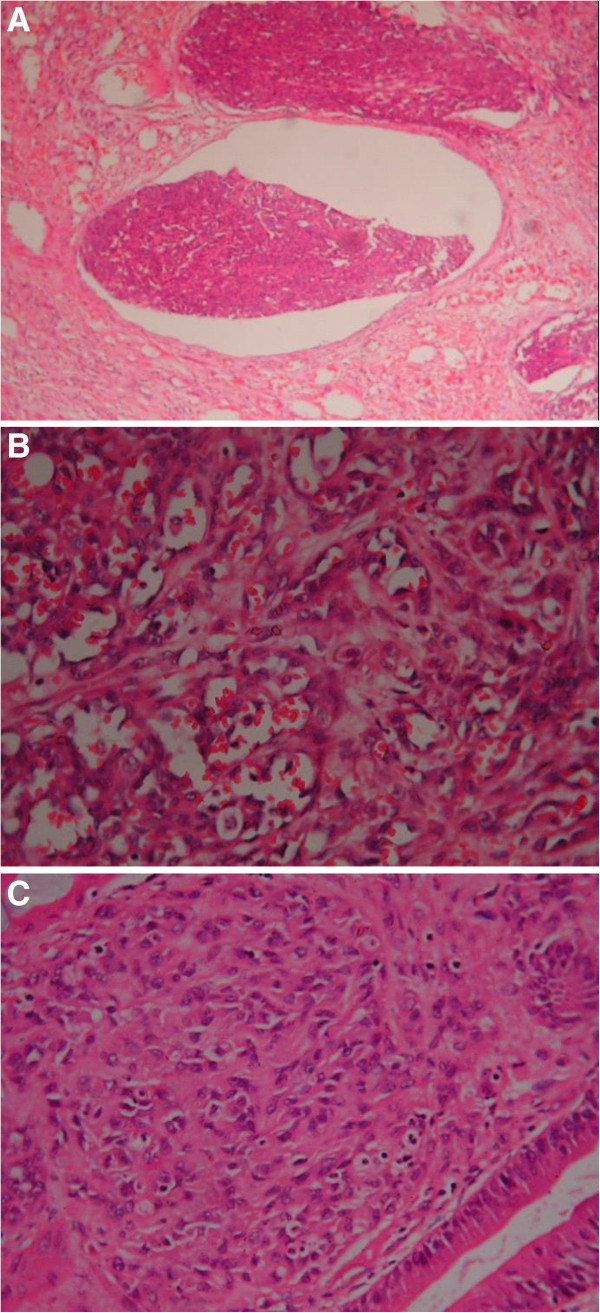
**Microscopic examination. (A)** Tumour tissue can be observed invading the vascular cavity (hematoxylin and eosin stain; ×100). **(B)** Tumor blood vessels were abundant, with the tumor cells surrounding them (hematoxylin and eosin stain; ×400). **(C)** The tumor cells were arranged in a slit-shaped pattern (hematoxylin and eosin stain; ×400).

**Figure 4:**
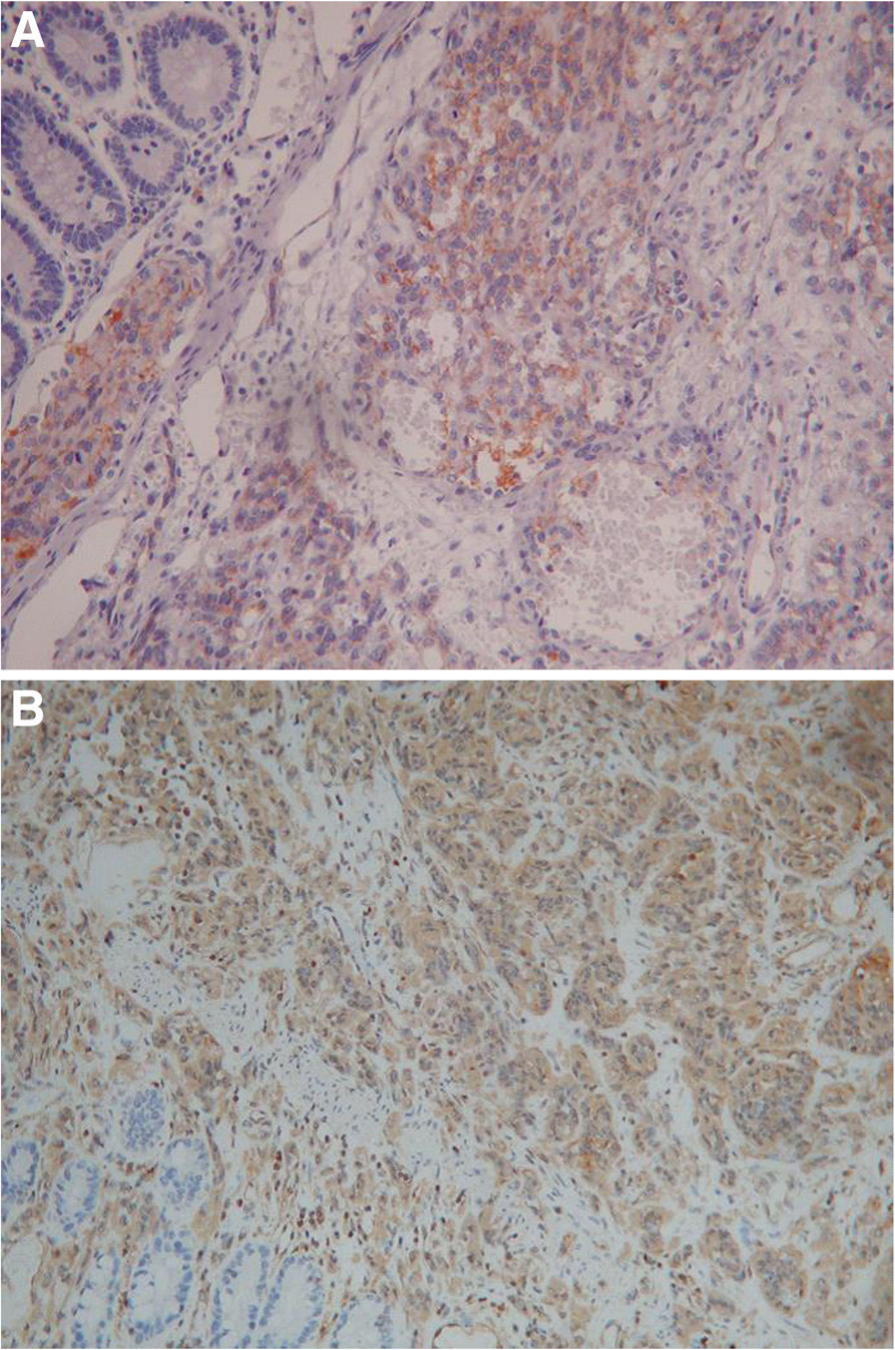
**Immunohistochemistry results.** The tumor cells were positive for **(A)** CD31 (hematoxylin and eosin stain; ×400) and **(B)** vimentin (hematoxylin and eosin stain; ×200).

An urgent general surgical consultation was required because of sudden severe aggravation of the abdominal pain. Abdominocentesis was performed, which showed a dark red fluid. The patient presented with peritonitis. Therefore, an emergency laparoscopic surgery was performed, which was later converted to a laparotomy following partial enterectomy. The operative findings included intra-abdominal bleeding of 1500 ml and an oedematous small intestine. Additionally, the greater omentum was found adherent to the small intestinal mesentery in the left upper abdomen with inflammation and blood oozing from the small intestine. During surgical exploration, dozens of irregular nodules of various sizes were found scattered on the surface of the liver. In addition, a mass of approximately 5.0 × 6.0 cm in size was found in the small intestine approximately 70 cm distal to the ligament of Treitz and was accompanied by an endoleak. During the operation, the tumor and the local mesentery of the small intestine were resected. Eight lymph nodes were examined, and two were found to be metastatic. On microscopic examination, vascular invasion of tumor tissue could be observed. The blood vessels of the tumor were abundant with tumor cells around them, and the tumor cells were arranged in a slit-shaped pattern (Figure 3). The immunohistochemistry results showed that the tumor cells were positive for CD31 and vimentin (Figure 4) and negative for CD34, actin, S-100, CD117 and CK56. Moreover, the Ki-67 proliferation index was less than 10% positive. On the basis of these findings, adenocarcinoma, intestinal tuberculosis, neuroendocrine tumor, malignant melanoma, Crohn’s disease, gastrointestinal stromal tumor (GIST) and lymphoma were excluded. Hence, the diagnosis of primary angiosarcoma of the small intestine with metastasis to the liver was confirmed.

After surgical resection, the patient was managed with adjuvant chemotherapy and palliative care. Approximately 400 to 500 ml hemorrhagic fluid was drained during the postoperative period through the abdominal drains. Moreover, the volume of hemorrhagic fluid increased to 600 ml on the 6th postoperative day. Therefore, a cytologic examination was performed, which showed malignant cells, confirming the diagnosis of malignant ascites. The patient received frequent blood transfusions due to a progressive drop in hemoglobin. In the early morning hours of the 27th postoperative day, the patient experienced a sudden disturbance of consciousness along with a progressive decrease in blood pressure. Due to the above-mentioned circumstances, further treatment was refused by the patient’s relatives. The patient died on the morning of the 27th postoperative day.

## Discussion

Angiosarcoma, a malignant neoplasm derived from the endothelial cells of blood vessels or lymphatic vessels, is characterized by aggressively proliferating and widely distributed tumor cells [6].

In 1879, Langhans and colleagues reported the first angiosarcoma in the spleen [7]. Thereafter, only a few cases of primary angiosarcoma involving the small intestine have been reported. Gentry and colleagues reviewed 106 vascular tumors of the gastrointestinal tract at the Mayo Clinic between 1925 and 1944, among which 16 angiosarcomas were found; furthermore, only three involved the small intestine [8]. Karpeh and colleagues reviewed 69 adult patients admitted to the Memorial Sloan-Kettering Cancer Center between 1982 and 1990. In this study, 35 angiosarcomas were found, and only 10 involved the viscus or retroperitoneum [9]. Naka and colleagues reviewed 99 Japanese patients with angiosarcoma diagnosed between 1974 and 1990, and no small intestinal angiosarcomas were found [3]. Allison and colleagues reviewed the cases of angiosarcoma that were diagnosed from 1990 to 2002 at the University of Washington and the Cleveland Clinic Foundation, finding only eight cases of angiosarcoma involving the gastrointestinal tract [10] (Table 1).

**Table 1:** Previous data regarding angiosarcoma and small intestinal angiosarcoma in the study population

**Reference**	**Study population**	**Number of patients with angiosarcoma**	**Number of patients with small intestinal angiosarcoma**
Gentry *et al*. [8] 1949	106 patients with vascular tumors of the gastrointestinal tract between 1925 and 1944.	16	3
Karpeh *et al*. [9] 1991	69 adult patients admitted to the Memorial Sloan-Kettering Cancer Center between 1982 and 1990.	35	≤10
Naka *et al*. [3] 1995	99 Japanese patients with angiosarcoma between 1974 and 1990.	99	0
Allison *et al*. [10] 2004	19 previously reported cases of angiosarcoma involving the gastrointestinal tract between 1990 and 2002.	19	8

To gain a deeper understanding of primary angiosarcoma of the small intestine, we searched the PubMed database. The following search terms were used: (“hemangiosarcoma”[MeSH Terms] OR “hemangiosarcoma”[All Fields] OR “angiosarcoma”[All Fields]) AND (“intestine, small”[MeSH Terms] OR “small intestine”[All Fields] OR “small bowel”[All Fields]) AND (English[Language]). Only 54 eligible articles were retrieved. We excluded articles that described secondary cases and articles describing animal angiosarcoma involving the small intestine. Finally, only 27 articles remained [1,5,11-35] (Table 2).

**Table 2:** Cases of primary angiosarcoma involving the small intestine reported in the English literature

**Authors**	**Sex/age (years)**	**Primary or secondary**	**Site**	**Immunohistochemical staining**	**History of prior radiation or other predisposing factor**	**Presentation**	**Treatment**	**Follow-up**
Maeyashiki *et al*. [11]	M/72	Indeterminate	Small bowel	Positive for CD31, CD34 and factor VIII	None	Anemia, melaena	Resection and daily blood transfusions	Died on hospital day 103
Siderits *et al*. [12]	M/79	Primary	Small bowel	Strongly positive for CD31	None	Obstruction	Resection	Unknown
Taxy and Battifora [13]	M/64	Primary	Small bowel	Positive for Factor VIII, collagen type IV and vimentin	Not available	Gastrointestinal bleeding	Resection	Died 1 year after the initial diagnosis
Taxy and Battifora [13]	F/57	Primary	Small bowel	Positive for Factor VIII, collagen type IV	Not available	Not available	Resection	Died shortly after surgery
Chami *et al*. [14]	M/59	Primary	Small bowel	Weakly positive for factor VIII-related antigen, Ulex europaeus I antigen and cytokeratin	None	Gastrointestinal bleeding, bowel obstruction, anorexiaand weight loss	Resection and transfusions	Died on the 11th day after surgery
Ordonez *et al*. [15]	M/80	Primary	Small bowel	Positive immunoreaction for FVIII-RAG	None	Anemia, undue tiredness and weakness	Resection	Died on the 20th postoperative day
Hwang *et al*. [16]	F/60	Primary	Small bowel	Positive for Ulex europaeus agglutinin 1	History of radiotherapy	Diffuse abdominal pain	Resection	Died 2 months after discharge
Mohammed *et al*. [5]	F/25	Primary	Small bowel	Not available	None	Intermittent abdominal pain, weight loss, abdominal distension, hematemesis and malaena	Resection	Died on the 11th day after surgery
Fraiman *et al*. [17]	M/85	Primary	Small bowel	Strong positivity for vimentin and CD31; focal positivity for factor VIII and CD34	None	Weight loss, anemia, weakness and abdominal pain	Resection and thalidomide	Not available
Selk *et al*. [18]	M/57	Primary	Small bowel	Not available	History of radiation therapy	Progressive abdominal distention and shortness of breath	Resection	Died 4 months after surgery
Berry *et al*. [19]	M/51	Primary	Small bowel	Positive for Ulex europaeus and vimentin	History of 3-year irradiation	Peritonitis	Resection, adriamycin and dacarbazine	Died 5 months after initial presentation
Watanabe *et al*. [20]	M/64	Primary	Duodenum and upper jejunum	Positive for vimentin and anti-endothelin-1	Not available	Persistent gastrointestinal bleeding	Not available	Died of pulmonary metastasis 1 year after the operation
Al Ali *et al*. [1]	M/87	Indeterminate	Small bowel	Positive for CD31	None	Lethargy, weakness and anemia	Resection	Died 6 weeks after the initial diagnosis
Khalil *et al*. [21]	M/68	Primary	Small bowel	Strongly positive for CD31, CD34 and vimentin	30 year history of heavy occupational exposure to radiation and polyvinyl chloride	Gastrointestinal bleeding and melaena	Resection	Died 6 months after initial presentation
Suzuki *et al*. [22]	F/61	Primary	Ileum	Positive for factor VIII-related antigen and Ulex europaeus agglutinin 1	20 year history of radiotherapy	Abdominal pain	Resection and intra-abdominal cisplatin	Died 1 year after initial presentation
Cilursu [23]	F/74	Indeterminate	Small bowel	Not available	Not available	Melaena	Resection	Not available
Delvaux *et al*. [24]	M/67	Primary	Small bowel	Positive for CD 31, CD 34, factor VIII-related antigen and keratin	Not available	Weight loss, intermittent severe abdominal pain and melaena	Resection	Died 3 months after diagnosis
Policarpio-Nicolas *et al*. [25]	F/51	Primary	Small bowel	Positive for CD 31, CD 34 and factor VIII-related antigen	History of irradiation	Abdominal pain	Resection	Died 10 months after laparotomy
Hansen *et al*. [26]	F/76	Primary	Small bowel	Positive for factor VIII and vimentin	History of irradiation	Watery diarrhea, vomiting, weight loss and abdominal pain	Resection	Died 5 months after operation
Aitola *et al*. [27]	F/50	Primary	Small bowel	Positive for CD 31, CD 34 and factor VIII-related antigen	≥10 year history of radiotherapy	Intestinal obstruction	Resection followed by combination chemotherapy with doxorubicin	1 year and 9 months after diagnosis, she was alive
Aitola *et al*. [27]	F/78	Primary	Jejunum	Positive for factor VIII-related antigen, CD31, CD34 and Ulex europaeus	≥10 year history of radiotherapy	Intestinal obstruction	Resection	Died of sepsis 2 years after diagnosis
Knop *et al*. [28]	M/72	Primary	Small bowel	Not available	Not available	Gastrointestinal bleeding and anemia	Resection	Not available
Ogawa *et al*. [29]	M/36	Primary	Small bowel	Positive for factor VIII-related antigen	Not available	Abdominal pain and nausea	Surgical treatment	Not available
de Mascarenhas-Saraiva *et al*. [30]	M/82	Primary	Ileum	Not available	Not available	Melaena and increasing shortness of breath	Surgical treatment and transfusions	Not available
Turan *et al*. [31]	Not available	Indeterminate	Jejunum	Not available	Not available	Acute abdominal signs	Resection and chemotherapy	Not available
Liu *et al*. [32]	F/39	Primary	Terminal ileum	Positive for CD31 and CD34	None	Increasing right iliac fossa pain, abdominal bloating and vomiting	Resection and chemotherapy	Not available
Kelemen *et al*. [33]	M/76	Primary	Small bowel	Positive for CD31	None	Abdominal pain and fatigue	Resection	Died of cardiac arrest on the 9th day after surgery
Fohrding *et al*. [34]	M/84	Primary	Small bowel	Positive for CD31, cytokeratin and vimentin; slightly weaker for CD34; Focally positive for factor VIII	Not available	Gastrointestinal bleeding	Resection, adjuvant chemotherapy with paclitaxel and transfusion	Not available
Grewal *et al*. [35]	M/73	Primary	Small bowel	Positive for CD31	None	Gastrointestinal bleeding, weakness and melaena	Resection	Died within 4 months of the diagnosis
M, male; F, female								

Only one younger case, a 25-year-old woman, was reported as having primary angiosarcoma of the small intestine with normal liver, spleen, rectum, urinary bladder, kidneys, uterus and adnexae [5]. To our knowledge, there are few reports of primary angiosarcoma of the small intestine with metastasis to the liver among such young patients in the English literature.

Intestinal angiosarcoma often presents with gastrointestinal bleeding, abdominal pain, intestinal obstruction, abdominal distention, weight loss, shortness of breath, anemia and weakness [5,10].

**Figure 5:**
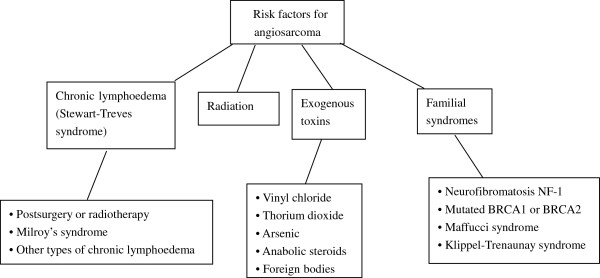
Risk factors for angiosarcoma.

Young and colleagues reviewed angiosarcoma with a focus on clinical trials and outlined its risk factors [2]. According to these authors, the risk factors for angiosarcoma were varied and are listed in Figure 5.

Magnetic resonance imaging (MRI), CT, positron emission tomography (PET), X-rays, ultrasound, endoscopy, immunohistochemical testing and a pathological examination can contribute to the diagnosis of angiosarcoma [2,30,31]. MRI, CT, abdominal X-rays and ultrasound are used to delineate the extent of the lesions in the preoperative period of abdominal angiosarcoma [2,31]. CT and PET may be helpful for detecting metastases in the preoperative period [2]. The lesions and the sources of bleeding can be detected by endoscopy. Meanwhile, endoscopic biopsy can be performed; significantly, wireless capsule endoscopy is a new endoscopic method that can help to improve the diagnosis of deep small intestinal pathology [30]. Von Willebrand factor, CD34, CD31, Ulex europaeus agglutinin 1, vascular endothelial growth factor, melanocytic markers (such as S100), human melanoma black-45, melanoma antigen and cytokeratins all are useful for diagnosis and differential diagnosis [2]. Moreover, poorly differentiated adenocarcinoma, intestinal tuberculosis, neuroendocrine tumor, malignant melanoma, Crohn’s disease, GIST, lymphoma and mesothelioma should be excluded, although with difficulty, in differential diagnosis [10,33]. Pathological and immunohistochemical examinations can contribute to the definitive diagnosis of angiosarcoma [21].

Due to the rarity of randomized trials and prospective studies, the management guidelines for other soft tissue sarcomas tend to be utilized when dealing with angiosarcoma [2]. Complete surgical excision tends to be impossible due to the aggressive proliferation and wide metastasis of angiosarcoma [1]. In the current setting, surgical excision associated with adjuvant radiotherapy and/or chemotherapy may be useful; however, the efficacy of these treatments for angiosarcoma remains unclear. Therefore, further studies are desperately needed to determine the optimal treatment for angiosarcoma [1].

Generally speaking, the prognosis of angiosarcoma is very poor. Moreover, several investigators have noted that the site of angiosarcoma tends to affect prognosis [2], and the prognosis of angiosarcoma of the small intestine is significantly worse than that of angiosarcoma at any other site [17]. Indeed, most patients die within a few months of diagnosis, and some die within the postoperative period [17]. Patients with intestinal angiosarcomas who survive over 1 year after diagnosis are extraordinarily rare [14].

## Conclusion

Primary angiosarcoma of the small intestine is an extremely rare and aggressive soft-tissue malignant tumor. The findings of this case report are of extreme significance. To our knowledge, this may be the first report of primary angiosarcoma of the small intestine with metastasis to the liver in such a young patient. In the future, we should focus on similar cases to ensure early diagnosis and proper treatment.

## Consent

Written informed consent was obtained from the patient for publication of this case report and any accompanying images. A copy of the written consent is available for review by the Editor-in-Chief of this journal.

## Abbreviations

CT: Computed tomography; GIST: Gastrointestinal stromal tumor; MRI: Magnetic resonance imaging; PET: Positron emission tomography.

## Competing interests

The authors declare that they have no competing interests.

## Authors’ contributions

QN searched the database, selected the articles, and wrote the manuscript. DS supervised the methodology, the selection of the articles, and the writing of the manuscript and is the corresponding author of the paper. HP assisted in drafting the manuscript. MR supervised the writing of the manuscript. WB, GL, XG and DS performed the surgeries. All the authors have read and approved the final manuscript.
